# Impact of Tobacco Smoke Exposure on Male Fertility: An In Vivo Study Using *Drosophila melanogaster*

**DOI:** 10.3390/cells14211689

**Published:** 2025-10-28

**Authors:** Natasha Gomes de Miranda, Ana Gajeiro, Ana Martins-Bessa, Isabel Gaivão

**Affiliations:** 1 Animal and Veterinary Research Center (CECAV), Associate Laboratory for Animal and Veterinary Sciences (AL4AnimalS), University of Trás-os-Montes and Alto Douro (UTAD), 5000-801 Vila Real, Portugal; al79678@alunos.utad.pt (N.G.d.M.);; 2 Department of Genetics and Biotechnology (DGB–ECVA), University of Trás-os-Montes and Alto Douro (UTAD), Quinta de Prados, 5001-801 Vila Real, Portugal; 3 Department of Veterinary Sciences (DCV-ECAV), University of Trás-os-Montes and Alto Douro (UTAD), Quinta de Prados, 5001-801 Vila Real, Portugal

**Keywords:** *Drosophila melanogaster*, tobacco smoke, male fertility, DNA damage, in vivo model, oxidative stress, genotoxicity

## Abstract

**Highlights:**

**What are the main findings?**

- Paternal tobacco smoke exposure significantly impairs the fertility, prolificacy, and longevity of offspring of two strains of *Drosophila melanogaster: Oregon K* (a wild-type strain proficient in all major DNA repair pathways) and *mus308* (a DNA repair-deficient strain).
- The DNA repair-deficient *mus308* strain showed higher vulnerability to spermatozoa cytotoxicity and a greater magnitude of reduction in spermatozoa count.

**What is the implication of the main finding?**

- The reproductive toxicity of tobacco smoke is genotype-dependent, suggesting that compromised DNA repair pathways exacerbate the transgenerational impact of environmental toxicants.
- *D. melanogaster* is a robust and sensitive in vivo model to mechanistically evaluate the paternal reproductive risks and transgenerational effects of complex smoke-related compounds.

**Abstract:**

Tobacco smoke has been consistently associated with impaired spermatozoa quality in men, including decreased concentration, motility, and increased morphological abnormalities. Key tobacco-related toxins such as nicotine and cadmium induce oxidative stress, leading to DNA damage in germ cells. This study aimed to evaluate the effects of tobacco smoke exposure on male fertility using *Drosophila melanogaster* as an in vivo model. Fertility, fecundity, parental toxicity, lifespan, and spermatozoa morphology were assessed in two strains: the wild-type *Oregon K* and the DNA repair-deficient *mus308*. Males were exposed to whole-tobacco smoke in a controlled environment for periods of 0, 1 and 7 min. Tobacco smoke exposure reduced fertility, fecundity, and offspring longevity in both strains. Additionally, spermatozoa from *mus308* males exhibited a higher frequency of morphological abnormalities. These findings demonstrate the detrimental impact of tobacco smoke on male reproductive function and suggest increased vulnerability in organisms with impaired DNA repair capacity.

## 1. Introduction

Infertility affects approximately 10–15% of human couples worldwide, with male factors accounting for nearly 20% of cases. Considering environmental factors, tobacco smoke exposure is robustly associated with detrimental impacts on sperm quality parameters, specifically manifesting as diminished concentration and motility, alongside elevated morphological abnormalities [1,2]. Tobacco (*Nicotiana tabacum*) contains numerous toxic components—such as nicotine and cadmium—that are known mutagens and carcinogens. These toxic components drive oxidative stress and subsequent DNA damage within germ cells, a pathway that ultimately compromises fertilization potential and elevates the risk of adverse reproductive outcomes [3,4,5].

The accumulation of tobacco smoke-derived toxic compounds is observed at concentrations in seminal fluid exceeding those found in the bloodstream, emphasizing a direct toxicological impact on spermatogenesis. Several studies have demonstrated a dose-dependent relationship between tobacco exposure and the severity of sperm impairment, because men who have smoked for more than five years have significantly worse seminal parameters than non-smokers or those who have smoked for less time [6,7,8]. Notably, cadmium has been implicated in increased oxidative stress, depletion of antioxidant defences, and dysfunction of sperm plasma membrane enzymes such as Ca^2+^-ATPase. Cadmium exposure also disrupts essential trace elements like zinc and magnesium, which are critical for DNA replication, chromatin stability, and overall sperm functionality [9,10,11,12].

The detrimental effects of tobacco smoke on male reproductive health have been extensively studied in mammalian models. However, research using dipteran species, particularly *Drosophila melanogaster*, remains limited despite their increasing use in toxicological and genotoxicity assays. Adopting *Drosophila melanogaster* as an in vivo model offers significant logistical benefits, including its accelerated life cycle, elevated reproductive output, and economical laboratory upkeep. The fruit fly *Drosophila melanogaster* is extensively employed in genotoxicity research because many of its genetic repair mechanisms and cellular pathways are evolutionarily conserved with those found in mammals. This similarity enables scientists to perform detailed mechanistic studies on toxic effects in a simpler and high-throughput model organism. Although *Drosophila* is useful for preliminary reproductive toxicity screening, its distinct reproductive physiology and absence of key mammalian pathways limit result extrapolation. Thus, complementary mammalian studies are essential for accurate assessment of EDCs and teratogens [5,13,14,15,16,17,18,19].

This study aimed to evaluate the effects of tobacco smoke exposure on male fertility using *D. melanogaster* in vivo. Additionally, the study sought to characterize changes in spermatozoa morphology, thereby contributing to the limited body of literature on *Drosophila* reproductive toxicology.

## 2. Materials and Methods

### 2.1. Strains

In *D. melanogaster*, the repair of DNA double-strand breaks (DSBs) predominantly relies on three established mechanisms: homologous recombination (HR), the classical non-homologous end joining (c-NHEJ) pathway, and the microhomology-mediated end joining (MMEJ) pathway. The present investigation utilized two distinct fly strains:

- *Oregon K* (*OK*): This strain serves as the wild-type control and is fully competent in processing DNA lesions across all primary repair mechanisms, specifically HR, c-NHEJ, and MMEJ [20,21,22].
- *mus308:* This line is homozygous for the *mus308* mutation, which results in a non-functional dmPolQ protein (the functional equivalent of human DNA polymerase θ, or PolQ). The *mus308* mutation imparts severe deficits in the repair of DNA interstrand cross-links and complex, persistent DNA damage. Its principal role is recognized in alternative end-joining pathways, particularly MMEJ, in addition to its involvement in translesion DNA synthesis and damage bypass [20,21].

### 2.2. Medium Conditions

All fly populations were maintained under carefully regulated laboratory conditions, including a controlled temperature and a consistent 12:12 h light–dark cycle. Under these standardized conditions, the species completes its life cycle in approximately 10–12 days, with adult flies living on average between 40 and 50 days. For colony maintenance, newly eclosed adults were transferred to clean glass containers supplied with the standard nutritional medium (Table 1). The culture vials were then securely closed using cotton plugs, a technique employed to facilitate necessary gas exchange while safeguarding against microbial contamination [22].

### 2.3. Fertility, Prolificacy, and Toxicity Assessment

Flies were divided into four experimental groups based on genotype and treatment: *Oregon K* control, *Oregon K* exposed to tobacco smoke, *mus308* control, and *mus308* exposed to tobacco smoke. Males and females aged between 0 and 1 days were selected. A total of 180 males were used, equally distributed between the *Oregon K* and *mus308* strains (90 males per strain). For the toxicity and longevity tests, 75 males were used per strain. For the fertility and prolificity tests, a total of 45 replicates were used per group, with one male per replicate.

Males in the treatment groups were exposed to tobacco smoke by being placed in a 600 mL glass beaker that was covered with a net. A lit Marlboro^®^ Gold cigarette butt was placed inside to initiate the exposure, creating a smoke concentration of approximately 1 mg/L. This specific brand was chosen because it is a low-yield cigarette (0.6 mg of nicotine, 6 mg of tar, and 7 mg of carbon monoxide under the ISO regime. These values increase dramatically under the Health Canada intense regime, as ventilation is blocked, allowing for more smoke delivery. The exposure consisted of three sequential intervals of 0, 1, and 7 min. This duration was determined through preliminary tests to be the maximum survival time for the flies under these conditions.

Following eclosion of the offspring, the number of adult males, females, and pupal deaths was recorded for each vial. Reproductive fitness (fertility) was quantified by the percentage of exposed males capable of successfully generating viable progeny. Reproductive output (prolificacy) was calculated as the aggregate count of offspring yielded from all tested males within a given cohort. Toxicity assessment was based on measuring the acute mortality rate observed in the exposed male population following the treatment phase. All trials were conducted using 3 independent biological replications, with 15 mating tubes constituting each experimental group.

### 2.4. Longevity Assay

Following the completion of progeny counts, the F_1_ offspring of each experimental condition (*Oregon K* control, 31 total offspring; *Oregon K* 1 min tobacco exposure, 32 total; *Oregon K*, 7 min tobacco exposure, 41 total; *mus308* control, 27 total; *mus308* 1 min tobacco exposure, 32 total; and *mus308* 7 min tobacco exposure, 41 total) were moved to 3 replicate vials corresponding to their respective strain and exposure group. All groups were subsequently housed in 200 mL glass vials, each containing approximately 25 mL of culture medium. To ensure a steady nutritional quality and prevent the build-up of pathogens or contamination from differing developmental stages, the flies were moved to fresh culture media every 7 to 9 days. The total duration in days, measured from adult eclosion until the time of death for every individual, defined longevity, with the final dataset incorporating individuals from both sexes [22].

### 2.5. Morphological Examination of Spermatozoa

One virgin male with *Oregon K* and *mus308* strains, aged 8–9 days, underwent tobacco smoke exposure for one minute before anesthesia via ethyl ether. Dissection of the seminal vesicles occurred in 10 μL of Ringer’s solution (130 mM NaCl, 35 mM KCl, 2 mM CaCl_2_; pH 6.5, adjusted with NaOH). The vesicles were subsequently moved to a second slide containing another 10 μL aliquot of fresh Ringer’s solution. Using a fine pin, the vesicles were carefully punctured to liberate the spermatozoa. Tweezers assisted in gently swirling the vesicle within the drop to enhance spermatozoa release [23]. Following this, 5 μL of the resultant spermatozoa suspension was combined with 20 μL of 1% low-melting-point (LMP) agarose (Pronadisa Micro & Molecular Biology, Madrid, Spain) pre-warmed to 37 °C. This final mixture was uniformly dispersed onto a microscope slide previously treated with 150 μL of 0.5% normal melting point (NMP) agarose (Sigma-Aldrich, St. Louis, MO, USA). An 18 × 18 mm coverslip was carefully seated over the preparation and pressed gently for gel flattening. To allow for solidification, the slides were placed at 4 °C for 5 min. After the coverslip was gingerly taken off, dehydration was achieved by sequentially immersing the slides in 70%, 90%, and 96% ethanol, followed by air-drying at room temperature. The process concluded with staining using 20 μL (1 μg/mL) of DAPI (4′,6-diamidino-2-phenylindole) to enable morphological assessment and counting of spermatozoa (1 male fly per slide from the post-treatment groups was evaluated), utilizing an Olympus BX61 (Olympus/Evident Scientific, Tokyo, Japan) fluorescence microscope at 200× magnification.

### 2.6. Statistical Analysis

For the longevity analysis, data were processed utilizing the OASIS 2 (Online Application for Survival Analysis 2) web-based platform. Survival curves were constructed employing the Kaplan–Meier approach, reporting 95% confidence intervals. Assessment of overall differences across the survival curves was carried out via the log-rank test (chi-square test), with corresponding *p*-values being documented. To establish the statistical variance separating the experimental treatment cohorts from the control group, an F-test was employed, which was executed immediately following the required analysis of variance (ANOVA) applied to the prolificacy data in its entirety. Statistical significance was designated at a threshold of *p* < 0.05.

## 3. Results

### 3.1. Male Fertility

Fertility was evaluated as the percentage of males producing at least one offspring per vial after exposure to tobacco smoke for 1 and 7 min in the *Oregon K* (*OK*) and *mus308* strains. The results are summarized in Table 2.

Exposure to tobacco smoke for 1 min significantly increased male fertility in the *OK* strain compared to the control group (87% vs. 60%, *p* < 0.05). In contrast, in the *mus308* strain, 1 min exposure resulted in a decrease in male fertility in the treated group relative to the control group (93% vs. 100%, *p* = 0.475); however, this difference was not statistically significant. After 7 min of exposure, male fertility in the *OK*-treated group increased to 100%, while in the *mus308-*treated group it decreased to 60%, with no significant differences.

#### 3.1.1. Prolificacy

**Figure 1:**
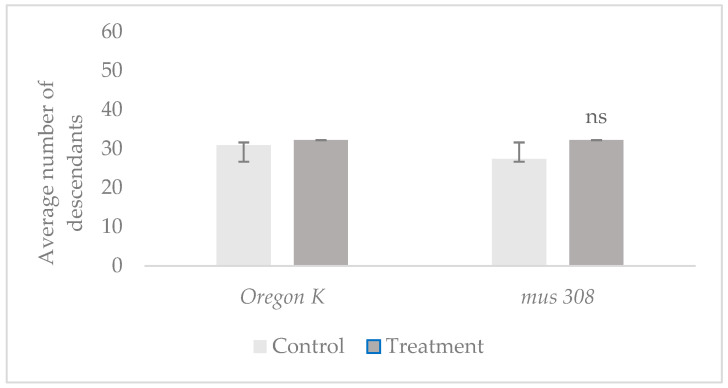
Mean number of offspring per tube in control and treatment groups of *Oregon K* and *mus308* strains after 1 min exposure to tobacco smoke. Error bars represent the standard deviation (*SD*). The 95% Confidence Interval (CI) is *OK* control [26.16, 35.68]; *OK* treatment [25.71, 38.65]; *mus308* control [23.15, 31.57]; and *mus308* treatment [27.95, 36.47]. The *p*-value is 0.473. The symbol (ns) indicates no statistically significant difference.

**Figure 2:**
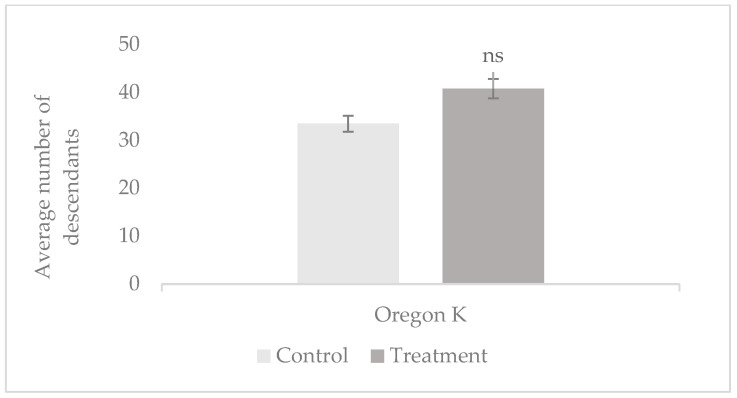
Mean number of offspring per tube in *Oregon K* and *mus308* strains after 7 min exposure to tobacco smoke. Error bars represent the standard deviation (*SD*). The 95% Confidence Interval (CI) for the mean number of offspring is control *OK* [24.04, 42.84]; treatment *OK* [27.35, 54.12]; control *mus308* [14.00, 29.87]; and treatment *mus308* [10.37, 28.52]. The symbol (ns) indicates no statistically significant difference.

Prolificacy was evaluated by measuring the average number of offspring per test tube in the control and treatment groups of both *Oregon K* (*OK*) and *mus308* strains. The 1 min tobacco smoke exposure experiments were based on 37 to 45 replicates per group using 37 to 45 males in total, while the 7 min exposures were based on 9 to 15 replicates per group using 15 males in total. The results are presented in Figure 1 and Figure 2.

Results indicate an increase in prolificacy in both strains following exposure to tobacco smoke. Specifically, in the *mus308* line (Figure 1), the number of descendants per tube was 32 (treatment) compared to 27 (control). For the *OK* line following 7 min exposure (Figure 2), the number of descendants was 41 (treatment) and 33 (control). These findings suggest that short-term exposure to tobacco smoke may stimulate an increase in prolificacy, particularly in the *Oregon K* strain, where the number of offspring per tube increased. A post hoc comparison between the *OK* control and *OK-*treated group showed a statistically significant difference (*p* = 0.034, d = 0.65). However, no significant differences were observed in the *mus308* strain (*p* = 0.473, d = 0.28, CI 95% = [−0.60, 1.16]) or in either strain after 7 min exposures (*p >* 0.05), indicating that the effect may be strain- and time-dependent.

#### 3.1.2. Male Toxicity

Toxicity was evaluated based on the mortality rate of males exposed to tobacco smoke for 1 min, as shown in the data, which are summarized in Table 3, based on a total of 75 males per strain exposed to tobacco smoke.

Exposure to tobacco smoke resulted in notable male mortality in both strains. The *mus308* strain showed a higher mortality rate (51%) compared to the *Oregon K* strain (40%), suggesting increased sensitivity to tobacco-induced toxicity. These results indicate that tobacco smoke exerts toxic effects on adult males with genotoxically compromised strains, like *mus308,* being more susceptible. No relevant mortality values were obtained in these studies in the control groups. No studies with the same methodology were found in the literature.

### 3.2. Offspring Longevity

**Figure 3:**
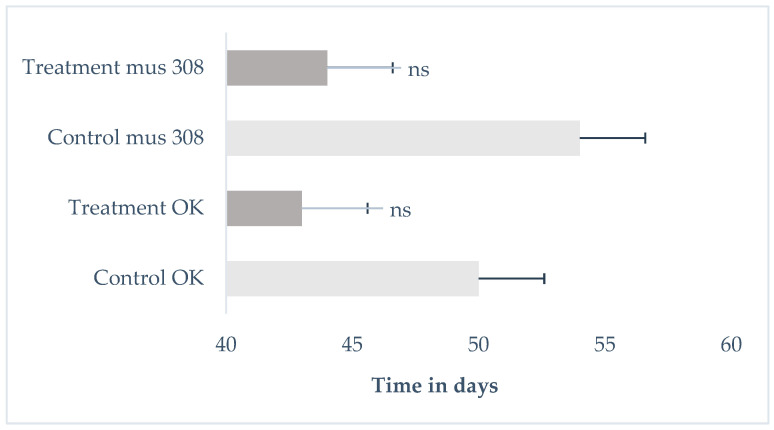
The average survival time (in days) of F_1_ male offspring from *Oregon K* (*OK*) and *mus308* strains after 1 min exposure to tobacco smoke. Error bars represent the standard deviation (*SD*). The 95% Confidence Interval (CI) is *OK* control [44.12, 55.88]; *OK* treatment [37.39, 48.61]; *mus308* control [48.45, 59.55]; and *mus308* treatment [39.12, 48.88]. The ANOVA *p*-value is *p* = 0.218 in the *OK* line and *p* = 0.072 in the *mus308* line. The symbol (ns) indicates no statistically significant difference.

**Figure 4:**
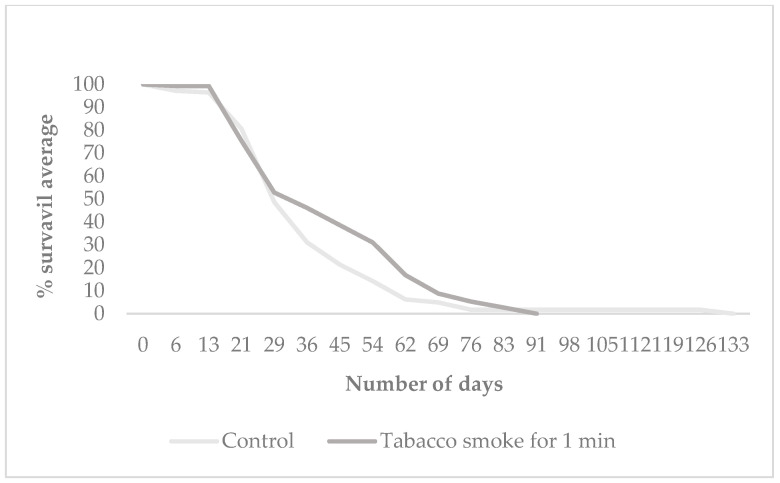
Kaplan–Meier survival curve of F_1_ offspring from the *Oregon K* strain (wild type) following 1 min exposure to tobacco smoke. A significant difference in the overall survival curve was found between the groups (Log-rank test: χ^2^ = 7.23, *p* = 0.007; Hazard Ratio (HR) = 1.34, CI 95% = [1.08, 1.66]).

**Figure 5:**
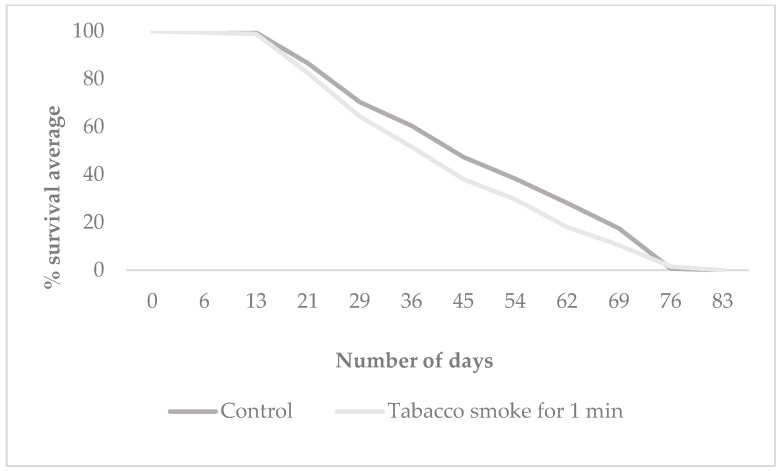
Kaplan–Meier survival curve of F_1_ offspring from the *mus308* strain following 1 min exposure of paternal males to tobacco smoke. A marked decrease in survival was observed in the treatment group, indicating increased vulnerability in DNA repair-deficient progeny. Statistical analysis was performed using the log-rank test. The value χ^2^ = 4.48 and *p*-value = 0.346.

Longevity was assessed by measuring the life expectancy of the F_1_ offspring exposed to tobacco smoke for 1 min, derived from the control and treatment groups *mus 308* and *O.K*. Figure 3 presents the mean survival time (in days) of F_1_ individuals from both *Oregon K* and *mus308* strains under control and tobacco smoke exposure conditions. Figure 4 and Figure 5 display Kaplan–Meier survival curves indicating the percentage of surviving F_1_ flies over time for each group. Each dataset represents the average of three independent replicates, totalling nine tests per condition, with 57 individuals on average per group.

Exposure to tobacco smoke resulted in a reduction in the mean lifespan of F_1_ offspring in both the *Oregon K* (*OK*) and *mus308* strains compared to their respective control groups. In the *OK* strain, the mean lifespan decreased from 50 days (control) to 43 days (treatment). A significant difference in survival was found between the groups (χ^2^ = 7.23, *p* = 0.007). Similarly, in the *mus308* strain, the mean lifespan was reduced from 54 days in the control group to 44 days following exposure. However, statistical analysis using the log-rank test for the *mus308* strain revealed a χ^2^ = 4.48 and a *p*-value = 0.346 indicating no significant difference in survival between the control and treated groups (HR = 1.25, CI 95% = [0.98, 1.60]). This suggests that the impact of paternal tobacco smoke exposure on offspring longevity is strain-dependent, with a significant effect on the overall survival curve observed only in the *Oregon K* strain. The difference in results between the mean lifespan (Figure 3) and the overall survival curve (Figure 4) reflects the power of the Log-rank test to detect time-dependent survival differences, even when the mean lifespans are not significantly different by ANOVA.

### 3.3. Spermatozoa Morphology

Spermatozoa morphology was analyzed using a modified version of the Single-Cell Gel Electrophoresis (SIM) technique as described by [23]. Samples were observed under a fluorescence microscope Olympus BX61 (Olympus/Evident Scientific, Tokyo, Japan, and images were captured using an integrated digital camera connected to the cellSens Standard software (Version 4.2). The number of spermatozoa was quantified on each slide, and morphological differences between the control and treatment groups were assessed qualitatively. An average total of 330 spermatozoa were counted on the slides analyzed for the *OK strain*, while 238 were counted on the slides analyzed for the *mus 308* strain, all from the control group.

**Figure 6:**
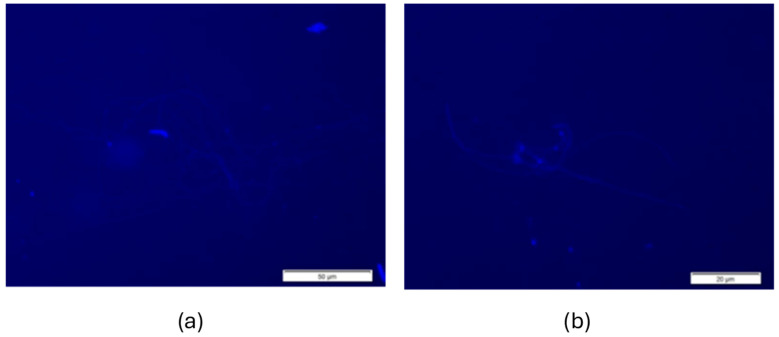
Fluorescence micrographs of spermatozoa cells from the *Oregon K* (*OK*) strain control group, stained with DAPI. (**a**): The spermatozoon presents a flat head and a long tail. (**b**) Spermatozoa present oval heads and long tails.

**Figure 7:**
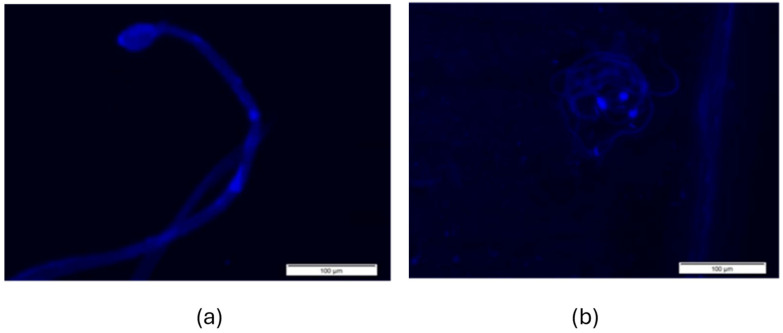
Fluorescence micrographs of spermatozoa from the *mus308* strain control group, stained with DAPI. (**a**): The spermatozoa are oval-headed and (**b**): the spermatozoa are oval-headed and are grouped together.

Figure 6 and Figure 7 display representative fluorescence micrographs of *D. melanogaster* spermatozoa from control groups, illustrating the typical morphology in the absence of exposure to tobacco smoke. Comparisons with treated groups were made to identify abnormalities potentially induced by tobacco smoke exposure.

Fluorescence microscopy revealed differences in the shape and organization of the spermatozoa head between the two strains. In the control group (Figure 6), spermatozoa from the *Drosophila OK* strain show no clusters and a flat head, which is normal for *Drosophila.* Figure 6a is representative of normal morphology, while Figure 6b shows a more oval-shaped spermatozoa head.

Figure 7a,b show that the spermatozoa of the *Drosophila mus308* control strain generally have a more oval-shaped spermatozoa head than the *OK* strain, even though it is a control group.

**Figure 8:**
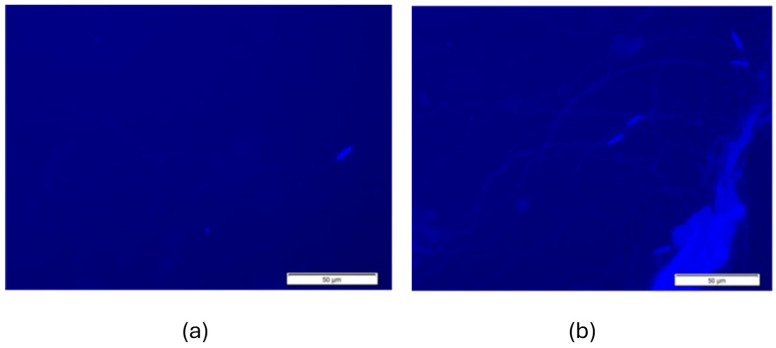
Fluorescence micrographs of spermatozoa from the *OK* lineage stained with DAPI in the tobacco smoke treatment group (1 min). (**a**) Isolated spermatozoa and (**b**): grouped spermatozoa.

**Figure 9:**
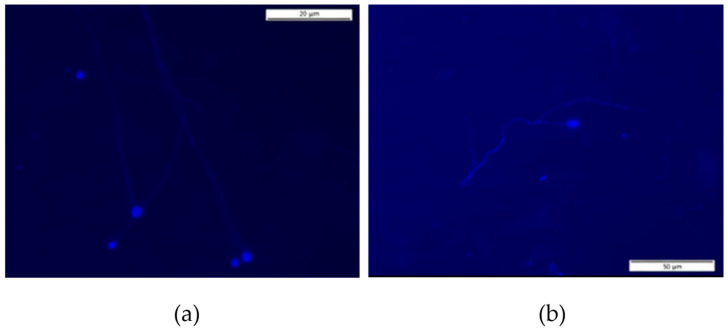
Fluorescence micrographs of spermatozoa of the *mus 308* strain stained with DAPI in the tobacco smoke treatment group (1 min). (**a**): The spermatozoa have rounded heads, and (**b**): the spermatozoa have oval heads.

Figure 8 and Figure 9 display representative fluorescence micrographs of *D. melanogaster* spermatozoa from the *Oregon K* and *mus308* strain, illustrating typical morphology in the exposure to tobacco smoke for 1 min.

Morphological changes were evident after 1 min of exposure to tobacco smoke. In Figure 8a,b of the *O.K* strain, the morphology is relatively preserved and similar to the control. In Figure 9 of the *mus308* strain, almost all heads (a) have a round shape. In Figure 9b, the spermatozoa still preserves an oval shape. Both treated strains exhibited a significant reduction in total spermatozoa count when compared to controls (163 in *OK* and 152 in *mus308* for the treated groups, compared to 330 and 238 in the control groups; *p* < 0.05). Specifically, the reduction in *OK* was statistically significant (*p* = 0.014, d = 0.81, CI 95% = [0.15, 1.47]), and in *mus308*, the reduction was highly significant (*p* = 0.002, d = 1.25, CI 95% = [0.49, 2.01])—suggesting a cytotoxic effect of smoke exposure on spermatogenesis or viability.

The observed spermatozoa abnormalities and reduction in cell numbers are in line with evidence from other species, including mammals, where exposure to tobacco has been associated with genomic instability, such as DNA strand breaks, altered chromatin organization, and structural defects in spermatozoa cells [3,24,25,26,27,28], *mus308* is particularly relevant, as it is involved in DNA repair via translesion synthesis, and its deficiency may exacerbate damage caused by genotoxic agents like tobacco smoke [28]. Similar effects have been reported in other genotoxicity assays in *Drosophila*, highlighting the model’s relevance in toxicological studies [27,28,29,30,31].

Following smoke exposure, *mus308* spermatozoa heads predominantly shifted to a rounded morphology, as seen in the fluorescence images. This transition from an oval to a rounded form may indicate compromised chromatin condensation or nuclear architecture, possibly due to oxidative stress induced by tobacco smoke. This observation is in line with previous findings demonstrating a relationship between oxidative damage and altered spermatozoa head shape in *Drosophila* and other model organisms [31].

The data suggest that tobacco smoke induces morphological and quantitative alterations in *Drosophila* spermatozoa, with the *mus308* strain displaying heightened sensitivity, likely due to impaired DNA repair capacity.

## 4. Discussion

The observed dose-dependent effect of tobacco smoke on male *Drosophila* fertility, where low-level (1 min) exposure increased fertility in *OK* flies while higher doses were inhibitory, is a phenomenon that may be interpreted as a hormetic response. This concept, historically linked to the Arndt–Schulz principle, suggests that low doses of a stressor can stimulate a biological process that is inhibited by higher doses. Given the limited dose–response resolution of the present study, our results should be considered as a possible hypothesis rather than a definitive conclusion, providing a basis for future research to further investigate if a hormetic effect is present [25,26,27,28].

The toxicological analysis confirmed tobacco smoke’s harmful effects, particularly in the *mus308* strain, which showed a higher male mortality rate (51%) compared to the *OK* strain (40%) following exposure. These findings are consistent with previous studies by Moratore et al. (2009), which demonstrated that aqueous tobacco extracts induce significant mortality in *D. melanogaster* (around 50% mortality at 10% tobacco concentration after 24 h) [32]. Other insect models, such as flour beetles and cabbage aphids, have similarly shown reduced viability and reproductive performance after tobacco exposure, reinforcing the compound’s broad-spectrum toxicity [5,28].

Regarding longevity, the observed decline in the mean lifespan of F_1_ offspring across both strains is consistent with the published findings. While Linford et al. (2013) and Sun et al. (2013) reported that *D. melanogaster* can live between 50 and 91 days under standard conditions, our control groups exhibited values near the lower end of this range [33,34]. In contrast, the treatment groups displayed significantly reduced lifespans, suggesting that even brief parental exposure to tobacco smoke adversely impacts progeny survival. In survival analyses, only the *mus308* offspring exhibited a statistically significant decrease in survival probability, suggesting that intact DNA repair confers resilience to progeny. This result is in strong agreement with the observations of Piper and Partridge (2016), who highlighted the high sensitivity of *D. melanogaster* lifespan to environmental stressors, such as xenobiotic exposure and diet [35,36,37,38].

Spermatozoa morphology analysis, conducted using a DAPI-based fluorescence microscopy protocol adapted from the standard Sperm Integrity Morphology (SIM) technique, revealed typical flagellar features previously described by Kanippayoor et al. (2013) and López-Fernández et al. (2007) [15,39]. Despite the limited number of studies employing this specific technique in *Drosophila*, our results corroborate existing knowledge regarding normal spermatozoa architecture and provide visual evidence of altered spermatozoa counts in the *mus308* strain following tobacco exposure. This strain, which is deficient in microhomology-mediated end joining (MMEJ) repair due to a mutation in the *mus308* gene (encoding DNA polymerase θ), demonstrated increased sensitivity, suggesting that defective DNA repair pathways may exacerbate the cytotoxic and reproductive impacts of tobacco [14,16,24,31].

To further investigate tobacco-induced genotoxicity, our previous work on human lymphocytes in the comet assay revealed a dose-dependent increase in DNA damage, particularly basal (non-oxidative) damage, with the highest levels observed at 100% tobacco concentration. Interestingly, oxidative DNA damage remained low across all treatment groups, indicating that tobacco’s genotoxicity may be driven primarily through mechanisms other than oxidative stress. These observations are in line with findings by Piperakis et al. (1998), who also reported basal and oxidative damage following tobacco exposure, although our results suggest a predominance of non-oxidative lesions [40]. This aligns with Kleinsasser et al. (2005), who demonstrated nicotine-induced genotoxicity in lymphocytes, further supporting the relevance of tobacco constituents as direct DNA-damaging agents [41].

Therefore, the present study corroborates the findings of recent studies which demonstrate that toxic compounds in tobacco smoke, including nicotine and polycyclic hydrocarbons, induce oxidative stress, DNA damage and apoptosis in various human cell lines, including reproductive and epithelial cells. In animal models such as rats and mice, exposure to tobacco has been associated with hormonal changes, mitochondrial dysfunction, systemic inflammation, and impaired reproductive function. These findings reinforce the harmful effects of smoking on fertility and cellular integrity, as well as its role in the development of chronic diseases [42,43,44,45,46,47,48,49,50,51].

The results discussed in the following study, such as spermatozoa quantification, have some limitations that must be considered, as it is a new method with a few promising results in spermatozoa from flies. Therefore, future investigations into improving morphological studies of spermatozoa in *D. melanogaster* as a practical model for investigating the reproductive effects of tobacco are necessary. Furthermore, adapting the comet assay to specifically evaluate genotoxic damage in *Drosophila* spermatozoa is a promising way to advance our understanding of tobacco-induced reproductive toxicity and quantify exposure to tobacco smoke. The results of the present study, particularly those concerning spermatozoa quantification and morphological analysis, have some limitations that must be considered. This is a new method with few promising results regarding spermatozoa from flies. Therefore, future investigations are necessary to improve quantitative and morphological studies of spermatozoa in *D. melanogaster* as a practical model for studying the reproductive effects of tobacco. Additionally, adapting the comet assay to evaluate genotoxic damage specifically in *Drosophila* spermatozoa is a promising approach to advancing our understanding of tobacco-induced reproductive toxicity and quantifying exposure to tobacco smoke.

## 5. Conclusions

The present study concludes that tobacco smoke exposure profoundly impacts male fertility by significantly altering key biological parameters, including fertility, fecundity, and offspring longevity in the *mus308* and *OK* strains of *D. melanogaster*. The two *mus 308* strains exhibited more pronounced morphological alterations in spermatozoa, indicating a higher susceptibility to the toxic effects of tobacco smoke compared to the *OK* strain. These data establish that tobacco smoke, even at sub-lethal concentrations, modulates essential biological processes like fertility and lifespan, a response contingent upon the organism’s genetic background and intrinsic DNA repair capacity. The *mus308* strain emerged as a sensitive genetic model for studying genotoxic stress. These insights highlight the utility of *D. melanogaster* as a valuable in vivo model for assessing environmental genotoxins and their transgenerational effects.

## Figures and Tables

**Table 1:** Constituents of the *D. melanogaster* standard medium.

Ingredients	Amount (per Litre Distilled Water)
Sugar	100 g
Agar-agar	12 g
Inactive yeast	100 g
Propionic acid	5 mL

**Table 2:** Percentage of fertile males after tobacco smoke exposure (1 min and 7 min) in *Oregon K* (*OK*) and *mus308* strains. The 95% Confidence Interval (CI) is reported for each percentage.

Groups	Exposure Time	% Fertile Males	IC 95%
Control *O.K*	0 min	60	[45.7, 74.3]
Treatment *O.K*	1 min	87	[77.2, 96.8]
Treatment *O.K*	7 min	100	[79.0, 100.0]
Control *mus308*	0 min	100	[92.0, 100.0]
Treatment *mus308*	1 min	93	[85.5, 100.0]
Treatment *mus308*	7 min	60	[35.2, 84.8]

**Table 3:** Percentage of male mortality following 1 min tobacco smoke exposure in *Oregon K* and *mus308* strains (treatment group). The 95% Confidence Interval (CI) is reported for each percentage. The table is based on a total of 75 males per strain exposed to tobacco smoke.

Strain	% of Male Mortality	IC 95%
*Oregon K*	40	[28.9%, 51.1%]
*mus 308*	51	[39.7%, 62.3%]

## Data Availability

The original contributions presented in this study are included in the article. Further inquiries can be directed to the corresponding authors.

## Abbreviations

The following abbreviations are used in this manuscript:

*OK*	*Oregon K*
DSBs	DNA double-strand breaks
HR	Homologous recombination
c-NHEJ	Classical non-homologous end joining
MMEJ	Microhomology-mediated end joining
NMP	Normal melting point
LMP	Low melting point
SIM	Inclusion sperm in microgels
DAPI	4′,6-diamidino-2-phenylindole
